# Self efficacy associated with regression from pregnancy-related pelvic girdle pain and low back pain following pregnancy

**DOI:** 10.1186/s12884-023-05393-z

**Published:** 2023-02-21

**Authors:** Dai Chunmei, Chen Yong, Gong Long, Tan Mingsheng, Li Hua, Yi Ping

**Affiliations:** 1 Obstetrics and Gynecology Department, Yi Chang Central People’s Hospital, The First College of Clinical Medical Science, China Three Gorges University, Yichang, Hubei China; 2 71282 Army Health Company, Baoding, Hebei China; 3 Department of orthopedic, Beijing Ji Shui Tan Hospital, 4th Clinical Hospital of Peking University, Beijing, 100035 China; 4 Department of Orthopedic, China-Japan Friendship Hospital, No. 2 Yinhuayuan East Street, Chaoyang, Beijing, 100029 China; 5 Department of Acupuncture, Dongfang Hospital, Beijing University of Chinese Medicine, Beijing, China

**Keywords:** Self efficacy, Pregnancy-related pelvic girdle pain, Pregnancy-related low back pain, Regression

## Abstract

**Background:**

Self-efficacy, one’s ability to deal with pain, disability, and other symptoms through self-management techniques, positively affect the quality of life in patients with chronic diseases. Pregnancy-related back pain is a common musculoskeletal disorder pre- and postnatally. Hence, the study aimed to determine whether self-efficacy is associated with the development of back pain during pregnancy.

**Methods:**

Between February 2020 and February 2021, a prospective case-control study was performed. Women with back pain were included. The self efficacy was assessed by the Chinese version of the General Self-efficacy Scale (GSES). Pregnancy-related back pain was measured using a self-reported scale. No regression from pregnancy-related back pain is defined as a recurrent or persistent pain score ≥ 3 over a week around 6 months postpartum. Women experiencing back pain during pregnancy are classified according to whether having a regression. This problem can be divided into pregnancy-related low back pain (LBP) and posterior girdle pain (PGP). The differences in variables were compared between groups.

**Results:**

A total of 112 subjects have completed the study finally. These patients were followed up with an average of 7.2 months after childbirth ranging from six to 8 months. 31 subjects (27.7%) of the included women did not report regression 6 months postpartum. The mean self efficacy was 25.2 (SD:10.6). Patients with no regression tended to be older (LBP:25.9 ± 7.2 vs.31.8 ± 7.9, *P* = 0.023; PGP: 27.2 ± 7.9 vs. 35.9 ± 11.6, *P* < 0.001*), have a lower self efficacy (LBP:24.2 ± 6.6 vs.17.7 ± 7.1, *P* = 0.007; PGP: 27.6 ± 6.8 vs. 22.5 ± 7.0, *P* = 0.010), and need high daily physical demand in their vocations (LBP:17.4% vs. 60.0%, *P* = 0.019; PGP: 10.3% vs. 43.8%, *P* = 0.006) when compared to those with regression. Multivariate logistic analysis shows that risk factors for no regression from pregnancy-related back pain included LBP (OR = 2.36, 95%CI = 1.67–5.52, *P* < 0.001), pain ratings of the onset of back pain during pregnancy≥3(OR = 2.23, 95%CI = 1.56–6.24, *P* = 0.004), low self efficacy (OR = 2.19, 95%CI = 1.47–6.01, *P* < 0.001), and high daily physical demand in their vocations (OR = 2.01, 95%CI = 1.25–6.87, *P* = 0.001).

**Conclusions:**

Low self efficacy makes the women experience about two-fold risk to experience no regression from pregnancy-related back pain. Evaluation for self efficacy is simple enough to be used to improve perinatal health.

## Introduction

Back pain during pregnancy is reported to be a relatively common musculoskeletal disorder during pregnancy and after childbirth, with an estimated incidence between 30 to 78% [1, 2]. At present, this problem is known as a multifactorial disease without definite etiology [3, 4]. Ostgaard et al. reported that 15% of the gravida dated the commencement of back pain to the time of their gestation [5]. Unfortunately, such pain may led to mental health issues, such as depression and anxiety, and restricted physical activity during pregnancy [3, 4, 6]. In cases with severe pregnancy-related back pain, these women would experience a disturbing quality of life, with limitations in activities of daily living and productivity at work [6]. Unfortunately, even with a series of treatments, regression of pregnancy-related back pain after delivery may occur [4, 6]. Previous studies showed the prevalence of back pain from the postpartum stage to 3 years and 6 years after childbirth is 43% [7] and 7% [8], respectively.

Back pain during pregnancy is considered highly heterogeneous. In general, this problem can be divided into two forms: pregnancy-related low back pain (LBP) and posterior girdle pain (PGP), according to the characteristics of symptoms, pain development, and physical examination [9–11]. The percentage of pregnancy-related LBP remains relatively constant at approximately 10% throughout gestation [11]. By contrast, 20% of women experience pregnancy-related PGP during pregnancy [12]. Additionally, following childbirth, regression patterns differ; pregnancy-related LBP frequently fails to regress postpatrum, whereas pregnancy-related PGP is usually a self-limiting condition, and symptoms generally resolve within a few weeks to a few months after delivery [10, 13, 14].

Recent studies have demonstrated the impact of cognitive-behavioral factors on chronic pain and/or functional disability in the general population [15, 16]. Self-efficacy is one’s ability to deal with pain, fatigue, disability, emotional distress, and other symptoms through self-management techniques. Higher self-efficacy has been demonstrated to positively affect mobility, activities of daily living, and quality of life in patients with many chronic diseases such as rheumatoid arthritis (RA) [17], chronic musculoskeletal pain [18], diabetes [19] and chronic obstructive pulmonary disease [20]. Limited self-efficacy could result in fear of movement and catastrophizing and may prevent recovery from chronic pain [18]. One study demonstrated that self-efficacy plays a crucial role in the perinatal period when women would experience a significant change physically and mentally [21]. However, there are few studies about the role of self-efficacy in different sub-type of pregnancy-related back pain. Therefore, the purpose of this study is to determine whether poor self-efficacy is associated with no regression of pregnancy-related LBP or PGP during/following pregnancy.

## Patients and methods

### Subjects

Between February 2020 and February 2021, a prospective case-control study was performed with the approval of the institutional review board of Yi Chang Central People’s Hospital. During this time, pregnant women who reported back pain were recruited at an obstetrics unit in the 12th week of pregnancy by research investigators. Subjects was recruited in the 12th week of pregnancy since this is generally the first scheduled appoint. Considering self efficacy is generally stable. Therefore, we measured the GSES at the time point. All the enrolled subjects granted written consent prior to beginning the study. Participants were able to withdraw their participation at any time point.

Inclusion criteria included ① women aged from 20 to 45 years old who were experiencing a healthy, non-complicated pregnancy; ② primiparous women; ③ rendered a diagnosis of back pain during the study period; ④ and no history of back pain prior to pregnancy.

Excluded criteria included ① a history of any disease before pregnancy or substance abuse; ② adverse life events during the present pregnancy such as stillborn fetus, severe fetal malformations; ③pregnant with multiples；④ unexpected abortion; ⑤ women with scoliosis, previous spine-related surgery and abdominal surgery；⑥and pregnancy via reproductive medicine.

### Data collection

#### Self efficacy

Self-efficacy is used to evaluate the person’s confidence to manage chronic diseases such as pain, emotional distress, and other symptoms using self-management abilities [15]. Self-efficacy was assessed using Chinese version of General Self-efficacy Scale (GSES) [22, 23], through an interview (or telephone call, if patient could not attned the hospital in person) in the 12th week of pregnancy. This scale included 10 items with a four-point likert scale from one (“*I can do nothing to protect myself from disease without confidence*”) to four (“*I can do a lot to protect myself from illness with total confidence*”). Individual item scores are summed for a total GSES score of 10–40. A higher overall score reflected a higher degree of self-efficacy. The scale has good reliability and validity [24].

Self efficacy level was dichotomized based on the mean value of 25 as the cut off points (see Table 1). The score below 25 were defined as low self efficacy that subjects had.

**Table 1:** Baseline characteristics of the included subjects, *n*=112

Clinical parameters	LBP	PGP	All
Subjects n (%)	38( 33.9%)	74 (66.1%)	112 (100%)
Age (years) mean (SD)	28.5±7.4	29.0±7.8	28.8±7.6
Pre-pregnant BMI (kg/m2) mean (SD)	23.7±3.2	23.4±3.6	23.5±3.4
Cigarette n(%)	6 (15.8%)	10 (13.5%)	10 (13.5%)
Educational Levels ≥university n (%)	25( 65.8%)	51 (68.9%)	76 (67.9%)
Self-efficacy mean (SD)	25.4±10.0	25.1±10.9	25.2±10.8
Onset of back pain (gestational weeks) mean (SD)	22.9±5.6	22.6±5.0	22.7±5.4
Pain rating at onset of back pain during pregnancy mean (SD)	3.8±2.0	3.5±2.0	3.6±2.1
Sick leave≥90 days n (%)	11 (28.9%)	23 (31.1%)	34 (30.4%)
High daily physical demand in their vocations n (%)	9 (23.7%)	17 (23.0%)	26 (23.2%)

*SD* Standard deviation, *BMI* Body mass index, *LBP* Low back pain, *PGP* Posterior pelvic pain

#### Pain assessment

Pregnancy-related back pain was measured using a self-reported numeric rating scale from 0 as “*No pain*” to 10 as the “*Worst possible pain*” [25] through an interview (or telephone call, if patient could attend the hospital in person) at two-time points: Time point 1 (T1): in the morning during the third trimester before delivery; Time point 2 (T2): in the morning 6 months after delivery.

#### Orthopedic testing

During the period of pregnancy and 6 months postnatally, all the women who experienced back pain would be referred to a multidisciplinary team, which included an obstetrician, orthopedist, and physiotherapist. Participants were tested each time they came in for their antenatal appointments (12–14 scheduled appoints throughout the duration of the pregnancy).

Pregnancy-related LBP was determined as continuous or recurrent dull pain for more than 1 week from the lumbar spine, which starts early in pregnancy [9, 26]. Pregnancy-related LBP was characterized as pain above the sacrum, in the lumbar spine, decreased range of motion, tenderness in the erector spine muscle, and a negative result on the posterior pelvic pain provocation test (4P test) [26]. Pregnancy-related PGP is experienced between the posterior iliac crest and the gluteal fold, particularly in the vicinity of the sacroiliac joints. The pain may radiate in the posterior thigh and can also occur in conjunction with/or separately in the symphysis. The diagnosis of PGP can be reached after exclusion of lumbar causes. The pain or functional disturbances in relation to PGP must be reproducible by positive results on the 4P test [12].

Based on the results and discomfort level, individualized treatment plans were developled and included education regarding anatomy and kinesiology, back-strengthening exercises, reducing physical activity, avoiding overloading the pelvis, physiotherapy, manipulation, yoga training, and/or acupuncture.

**Fig. 1:**
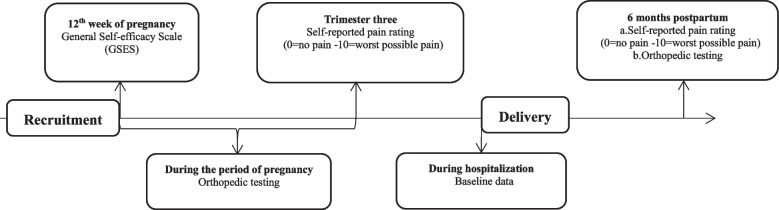
A flowchart about when each measure was performed

No regression from pregnancy-related LBP or PGP is defined as a recurrent or persistent pain score ≥ 3 over a week around 6 months postpartum, as the previous research has shown that pregnancy-related back pain significantly improves by this time point [10]. At 6-months following delivery, participants were asked to report to the outpatient clinic for clinical examination involving lumbar spine range of motion, palpation of lumbar paraspinal muscles, and 4P test by an experienced doctor. A flowchart about when each measure was performed is shown in Fig. 1.

#### Baseline data

Baseline data was collected via a survey and included demographic data (such as age, BMI, and educational level), the onset time of back pain (gestational weeks), the pain ratings at the onset of back pain during pregnancy, sick leave≥90 days, and daily physical demand levels in their vocations were asked through an electronically distributed questionnaire and recorded for the analysis. Baseline data were collected when they were admitted for delivery.

The onset time of back pain was dichotomized based on the 25 weeks as the cut off points as it was the scheduled appoint time which was closet to the mean onset of back pain (about 22 week in the present study, see Table 1).

High daily physical demand levels in their vocations was defined when a subject reported that she need daily frequent twisting/lifting movements in their vocations .

### Statistical analysis

All data were collected and analyzed using SPSS for Windows version 18.0 (SPSS Inc., Chicago, Illinois). Discontinuous data were demonstrated as percentages and compared via the Chi-square test. The Kolmogorov-Smirnov test was performed to determine the distribution of continuous data, whether mean ± standard deviation (SD) for normal distribution or the median and semi-interquartile range for non-normal distribution, respectively. A t-test or Mann-Whitney U was performed to compare the difference in variables between groups. The internal consistency reliability was measured using Cronbach’s alpha: previous studies suggest that Cronbach’s alpha > 0.5 is considered acceptable reliability [27, 28]. The intraclass correlation coefficient (ICC) was used to examine the one-week test-retest reliability of Chinese version GSES questionnaire. Acceptable test-retest reliability was greater than 0.75 [29]. Univariable and multivariate logistic regression analysis was performed to estimate the odds ratio (OR) and the associated 95% confidence interval (CI) to determine factors independently associated with poor prognosis of pregnancy-related back pain. A partial correlation analysis with age, BMI, high daily physical demand level in their vocations, and educational levels≥university as control variables was performed. The correlation was considered“*strong*”if r ≥ 0.5,“medium”if 0.5 > *r* ≥ 0.3, or“*weak*”if 0.3 > *r* ≥ 0.1. *P* < 0.05 was set as statistically significant and power analysis was≤0.9.

## Results

### Patient demographics

In total, 112 out of 125 women who responded to this study were eligible to participate. Of those excluded, five participants had a history of any disease before pregnancy or substance abuse; four had adverse life events during the present pregnancy such as a stillborn fetus; two utilized reproductive medicine to conceive; and the fetuses of two participants were severely malformed, and a resultant termination.

Baseline demographics can be found in Table 1. Briefly, 38 patients (33.9%) experienced pregnancy-related LBP, and the remainder of the participants pregnancy-related PGP. Patients were followed up at an average of 7.2 months (6–8 months) after childbirth and 27.7% of the included participants did not report regression of pain at 6 months postpartum. The mean GSES score for evaluating self efficacy was 25.2 (SD:10.6). Of the patients having a self efficacy score below the average level, 15 (48.4%) patients were experiencing LBP, and 16 patients (51.6%) were experiencing pregnancy-related PGP.

### Comparative analysis of clinical outcomes

Regardless of pregnancy-related LBP or PGP, patients with regression tended to be older (LBP: 25.9 ± 7.2 vs.31.8 ± 7.9, *P* = 0.023; PGP: 27.2 ± 7.9 vs. 35.9 ± 11.6, *P* < 0.001*), have a higher self efficacy level (LBP:24.2 ± 6.6 vs.17.7 ± 7.1, *P* = 0.007; PGP: 27.6 ± 6.8 vs. 22.5 ± 7.0, *P =* 0.010), and have high daily physical demand in their vocation (LBP:17.4% vs. 60.0%, *P* = 0.019; PGP: 10.3% vs. 43.8%, *P* = 0.006) when compared to those without regression. Specifically, those respondents who experienced pregnancy-related LBP with no regression after childbirth tended to have an earlier onset time of back pain (17.7 ± 5.9 vs. 27.3 ± 5.8 gestational week, *P* < 0.001) and stronger pain ratings of the onset of back pain during pregnancy (4.9 ± 2.4 vs. 2.7 ± 2.2, *P* = 0.006) with a higher incidence of sick leave≥90 days (73.3% vs.21.7%, *P* = 0.007) than those with regression (Table 2).

**Table 2:** Comparisons about the characteristics of back pain subjects between regression and no regression, *n*=112

	LBP			PGP		
	Regression	No Regression	***P*** value	Regression	No Regression	***P*** value
Subject n (%)	23 (60.5)	15 (39.5)	-	58 (78.4)	16 (21.6)	-
Age (years)	25.9±7.2	31.8±7.9	0.023*	27.2±7.9	35.9±11.6	<0.001*
Pre-pregnancy BMI (kg/m2)	24.0±3.8	24.5±3.4	0.682	23.2±3.2	22.9±3.5	0.746
Cigarette	5 (21.7)	4 (26.6)	0.967	5 (8.6)	2 (12.5)	0.990
Onset time of back pain (gestational weeks)	27.3±5.8	17.7±5.9	<0.001*	21.9±5.2	23.6±5.6	0.259
Pain rating at onset of back pain	2.7±2.2	4.9±2.4	0.006*	3.7±2.2	3.3±2.3	0.526
Educational levels ≥university	15 (65.2%)	8( 53.3%)	0.694	42 (72.4%)	11 (68.8%)	0.980
Sick leave ≥ 90 days	5 (21.7%)	11 (73.3%)	0.002*	14 (24.1%)	4 (25.0%)	0.797
Self-efficacy	24.2±6.6	17.7±7.1	0.007*	27.6±6.8	22.5±7.0	0.010*
High daily physical demand in their vocations	4 (17.4%)	9 (60.0%)	0.019*	6 (10.3%)	7 (43.8%)	0.006*

Mean ± SD or n(%) was shown. *BMI* Body mass index, *LBP* Low back pain, *PGP* Posterior pelvic pain.* Significant difference between groups

### Multivariate analysis of no regression from pregnancy-related Back pain

After controlling for age, multivariate logistic analysis shows that risk factors for no regression from pregnancy-related back pain included the type of pregnancy-related LBP (OR = 2.36, 95%CI = 1.67–5.52, *P* < 0.001), pain ratings at the onset of back pain during pregnancy≥3(OR = 2.23, 95%CI = 1.56–6.24, *P* = 0.004), low self efficacy (OR = 2.19, 95%CI = 1.47–6.01, *P* < 0.001), and high daily physical demand in their vocation (OR = 2.01, 95%CI = 1.25–6.87, *P* = 0.001) (Table 3).

**Table 3:** Risk factors for low back pain women without regression

Clinical parameters	Non-adjusted OR(95% CI)	***P*** value	Adjusted OR^a^ (95% CI)	***P*** value	Adjusted OR^b^ (95% CI)	***P*** value
LBP (vs. PGP)	2.23(1.54-5.40)	<0.001*	2.36(1.67-5.52)	<0.001*	2.44(1.75-5.59)	0.003*
The onset time of back pain (earlier than 25th week)	1.50(1.12-4.78)	0.172	1.65(1.28-4.93)	0.263	1.77(1.40-5.08)	0.182
Pain rating at onset of back pain during pregnancy ≥ 3	2.12(1.43-6.10)	0.002*	2.23(1.56-6.24)	0.004*	2.35(1.69-6.38)	0.002*
Low self-efficacy	2.10(1.38-5.87)	<0.001*	2.19(1.47-6.01)	<0.001*	2.34 (1.62-6.16)	<0.001*
Sick leave ≥ 90 days	1.62(1.21-5.00)	0.135	1.76(1.35-5.23)	0.152	-	-
High daily physical demand in their vocations	1.87(1.14-6.73)	0.003*	2.01(1.25-6.87)	0.001*	-	-

^a^Model I adjusted for age and pre-pregnant BMI. ^b^Model II adjusted for age, pre-pregnancy BMI, cigarette, educational levels ≥university, high physical demand in their vocations and sick leave≥90 days

*OR* Odds Ratio, *CI* Confidence interval, *BMI* Body mass index, *LBP* Low back pain, *PGP* Posterior pelvic pain

* Significant difference

### Correlation analysis between self efficacy level and pain scores at the last follow-up

A partial correlation test by controlling demographic factors, including age, BMI, high daily physical demand in their vocations, and educational levels ≥university, was performed to determine the correlation between self efficacy level and pain scores at the last follow-up. Self-efficacy had a positive correlation with pain scores (*r* = 0.583, *P* < 0.001).

### Reliability

The intraclass correlation coefficient (ICC) for the self efficacy questionnaire ranged from 0.86 to 0.93. Cronbach’s lumbar pain coefficient was 0.82, and that, for each item, ranged from 0.84 to 0.92.

## Discussion

The purpose of this study is to determine determine whether poor self-efficacy is associated with no regression of pregnancy-related LBP or PGP during/following pregnancy. The main findings showed that those with low self-efficacy during pregnancy tended to have no regression of back pain at 6 months postpartum. The multivariate logistic analysis showed low self-efficacy was associated with about a two-fold (OR = 2.19) increased risk of no regression from pregnancy-related back pain compared to high self-efficacy.

The relationship between self-efficacy and two types of pregnancy-related back pain have never been investigated before. However, the cross-sectional correlations between self-efficacy and chornic low back pain in the general population have already been examined in previous studies [27–29]. Ferrari et al. reported that self-efficacy displayed moderate correlations with pain intensity (*r* = − 0.41) and disability (*r* = − 0.55) after investigating 103 adult outpatients with nonspecific chronic low back pain [27]. The other cross-sectional study with a sample of 215 individuals with back pain showed that reduced self-efficacy is related to an increased level of functional disability [28]. A prospective, single-center orthopedic spine clinic demonstrated that self-efficacy was strongly negatively correlated with neck pain-related (*r* = − 0.69) and back pain-related (*r* = − 0.62) functional scores [29]. These studies supported self-efficacy in predicting back pain levels in the general population using consistent result. However, they failed to investigate the association between self efficacy and whether having regression of chronic low back pain. Psychological variables are more easily involved in developing pregnancy-related back pain than the general population [30–32]. Previous studies reported pregnant women with a high sense of self-efficacy may have a better ability to prepare for new-onset problems, find new interests, and invest in recent changes in such a particular period to adapt to various physical and psychological discomforts and environmental changes [33, 34]. All these positive minds contribute to producing a self-drive towards comprehensive health behaviors to deal with back pain problems. In other words, women who have high-level self efficacy generally do better in self-management and own more strengthened health awareness. For pregnant women who generally encounter both psychological and physiological difficulties, high self-efficacy tend to lead to a positive adaptation following stressful situations [35, 36]. Therefore, the present results may confirm that patients with a high self efficacy seemingly have an increased odds of recovering from low back pain.

Previous studies regarding the association between chronic pain and self-efficacy in the general population used the pain self-efficacy questionnaire (PSEQ) to quantify the levels of self-efficacy [28, 29]. However, considering more complex situations in pregnancy, such as high odds of anxiety and depression [30], more symptoms, and changed social roles may make PSEQ less reliable to reflect the actual situation. Instead, a modified version of GSES was used to evaluate the self efficacy with the expectation to reflect it more comprehensively. The present reliability analysis showed it has a good consistency.

The present study demonstrated that compared to PGP, LBP have increased risk for no regression from the pain, which was consistent with previous results [11, 14, 26]. Ostgaard indicated that after childbirth, the regression of these two pain types differs substantially. LBP does not regress as expected, whereas PGP diminishes at approximately 11 weeks postpartum [10]. Our results are mostly consistent with the previous study concerning that the pain ratings of the onset of back pain during pregnancy and the onset time in patients without regression from back pain was more intense and early compared to patients with recovery [7]. Of note, we noticed that such a difference was seen in the patients with the types of LBP.

### Strength and limitations

This study has strengths that should be mentioned. To the best of our knowledge, this is the first study assessing the predictive value of self efficacy for the prognosis of pregnancy-related low back pain. Regardless of pain experienced (LBP or PGP), patients with a higher self-efficacy level tended to regress following birth. After identifying the bad prognosis from back pain during pregnancy, it is helpful to improve functional recovery and prevent the debilitating consequence by the initiation of specific measures such as psychological interventions. Evaluation for self efficacy is simple enough to be used during the prenatal consultation. Hence, the predictive value of self efficacy evaluated in the clinical setting is of great necessity.

There were a few limitations associated with the the current study. First, on account of not increasing the subject burden, we merely evaluate the pain ratings during the 3rd trimesters of pregnancy and at about 6 months after childbirth to determine whether the regression for low back pain occurred. Whether regression or not depends on this two time points. Therefore, this may not reflect the actual progression of back pain. Second, the sample size was small and all were recruited from a single institution which may not be representative of a wider population. A larger sample from multiple centers was needed to support the present findings.

## Conclusion

Regardless of pain experienced during pregnancy, patients with regression of pain following birth tended to have a higher self-efficacy level compared to those without regression. Evaluation for self efficacy is simple enough to be used in the clinical setting to improve the pregnant woman’s health.

## Data Availability

Data can be made available upon request to the corresponding author.
